# Incivility and Knowledge Hiding in Academia: Mediating Role of Interpersonal Distrust and Rumination

**DOI:** 10.3389/fpsyg.2021.769282

**Published:** 2022-01-03

**Authors:** Qingyan Wu, Shahnawaz Saqib, Jianhua Sun, Yuxia Xiao, Wenya Ma

**Affiliations:** ^1^School of Marxism, Shenyang Jianzhu University, Shenyang, China; ^2^Department of Management Sciences, Khwaja Fareed University of Engineering and Information Technology, Rahim Yar Khan, Pakistan; ^3^Human Resource Center, Beijing Huaxia Lihong Commodity Inspection Co., Ltd., Beijing, China; ^4^School of Mathematics and Statistics, Wuhan University, Wuhan, China; ^5^Psychological Science (Conversion), University of Glasgow, Glasgow, United Kingdom

**Keywords:** workplace incivility, knowledge hiding, distrust, academia, rumination

## Abstract

Workplace incivility is under investigation for the last three decades, and it holds a central position in organizational behavior literature. However, despite the extensive investigations in the past, there exists a missing link between workplace incivility and knowledge hiding in academia. This study aims to tap this missing link for which data were collected from the universities staff. Data were collected in two waves to reduce the common method biases. In the first wave, questions were asked from the respondents regarding their demographic characteristics and exposure to workplace incivility. At this stage, 400 questionnaires were floated and 355 completely filled responses were received back, while in the second wave, those respondents were approached for data collection who have completely filled questionnaires in the first wave. The time interval between the two waves was 1 month. In the second wave, questions related to distrust and knowledge hiding behavior were asked from the respondents. At this stage, 323 questionnaires were received back out of which 290 were filled and these were considered for final data analysis. Collected data were analyzed by applying structural equation modeling (SEM) through SmartPLS. Results indicated that employees tend to hide knowledge when they experience incivility at workplace. Moreover, they develop a sense of distrust in response to workplace incivility which further triggers them to hide knowledge. Limitations and future directions are also discussed.

## Introduction

Workplace incivility is a prevalent phenomenon found in diverse cultures and organizations (Cortina et al., 2001; Schilpzand et al., 2014). Among different types of deviant behaviors, workplace incivility is the most hazardous for individuals/organizations (Andersson and Pearson, 1999; Blau and Andersson, 2005). It has been defined as “The low-intensity deviant behavior with ambiguous intent to harm the target, in violation of workplace norms of mutual respect” (Williams and Anderson, 1991). Such low-intensity counterproductive behaviors result in a heavy direct and indirect costs for the organizations (Porath and Pearson, 2013). The existing body of literature provides sufficient evidence regarding the toxic impacts of incivility for both, individuals and organizations (Schilpzand et al., 2014). Past studies indicate that these hazardous and toxic impacts of incivility decrease citizenship behavior (Dalal, 2005) and increase turnover rate (Chiaburu and Harrison, 2008). Incivility also induces stress among individuals (Bowling and Beehr, 2006), reduces work engagement, and lessens job satisfaction (Miner-Rubino and Reed, 2010; Giumetti et al., 2013). While in the case of the spillover effects of incivility, some researchers have documented lower marital satisfaction accompanied by work-family conflicts (Lim and Lee, 2011; Ferguson, 2012).

Despite extensive studies in the past to curb incivility at the workplace, there is still a rising number of complaints by individuals regarding exposure to incivility at the workplace (Porath, 2016). Uncivil behaviors prevail commonly within organizational circuits, and usually, 90 % of employees experience it within office space (Lim and Lee, 2011). Thus, the rising incidence of incivility indicates that there is a need to mitigate its harmful effects (Burnes and Pope, 2007; Griffin, 2010) because costs associated with workplace incivility are to be tolerated by employees and originations simultaneously.

The concept of incivility is defined as an action that is ill-mannered, impolite, irritated, and demonstrating a gesture of lack of respect (Kane and Montgomery, 1998). Such behaviors trigger victims to respond by attacking the perpetrator or simply releasing frustration on the other colleagues which creates a hostile work environment. In some cases, perpetrators are not in a position to attack back due to lack of power which breeds a sense of humiliation and shame among them and they tend to involve in counterproductive behaviors. This involvement might be a strategy to manage personal resources to cope up with a sense of humiliation (Porath and Pearson, 2013).

Nevertheless, what is the medium to release the frustration caused by incivility, it has negative consequences for victims, and it triggers negative emotions among them (Porath and Pearson, 2012), such state filled with negative emotions motivates employees to showcase different self-defensive behaviors in the future to cope the poisonous effects of workplace incivility (Sears and Humiston, 2015).

Although workplace incivility has been investigated in depth by researchers in the past (Saqib et al., 2017; Sarwar et al., 2019; Bashir et al., 2020) and it is a well-documented phenomenon, there is still need to investigate it in public sector organization. Previous research has demonstrated how workplace incivility contributes to knowledge hiding behaviors (Connelly et al., 2012; Peng, 2013); however, there seems a missing link that how in academic institutes employees tend to cope with workplace incivility by showing discretionary behaviors in the shape of knowledge hiding (Srivastava et al., 2006).

Previous studies have recommended investigating the role of workplace incivility and knowledge hiding through other counterproductive behaviors, and thus, this study has anticipated the future call of Irum et al. (2020) by assuming that employee rumination and interpersonal distrust can stem as negative affect in response to workplace incivility. Moreover, as suggested by Irum et al. (2020), this study empirically tested the relationship between workplace incivility and knowledge hiding by collecting the data from employees of public sector universities.

Thus, this study sought to contribute from many perspectives; firstly, from a theoretical perspective, this study has established a new link into the literature by testing the relationship of rumination and knowledge hiding; secondly, relationship between workplace incivility and knowledge hiding has been tested by collecting the data from employees of public sector universities (Irum et al., 2020); and thirdly, data have been collected from those faculty members who were supervising research students of various degree programs at the time of data collection; thus, this study also adds into the body of knowledge that knowledge hiding in higher education institutes can block the flow of knowledge that will eventually influence the individual and organizational performance (Tangaraja et al., 2015; Millar et al., 2016).

### Hypotheses Development

Workplace incivility was developed as a central subject in the literature of organizational behavior during the last three decades. A plethora of research has investigated that how an organization, group, and individual-level outcomes are influenced by incivility and its related phenomena, such as workplace deviance, bullying, and aggression. Workplace incivility is defined as “low-concentration deviant behavior with unclear intention to harm the victim in the destruction of workplace norms for common respect” (Andersson and Pearson, 1999). Workplace incivility is common but its effects are not. Job stress, cognitive distraction, psychological distress, lower job satisfaction, and greater turnover intention as a result of high uncivil behavior have been documented (Pearson et al., 2001; Cortina et al., 2002; Lim et al., 2008). The conservation of resource (COR) theory supports the premise of this study and explains how individuals deal with valuable resources to be preserved in response to counterproductive work behaviors such as incivility (Hashemi et al., 2018). Moreover, as a result of hostile and uncivilized treatment at the workplace, interpersonal distrust emerges which limits the development of new relationships and ultimately block resource creation (COR; Agarwal et al., 2021). Another perspective in this study is based on the “desperation principle,” which leads employees toward a defensive mode and individuals tend to safeguard their resources from depletion by keeping quiet (Holmgreen et al., 2017; Agarwal et al., 2021).

### Workplace Incivility, Rumination, and Knowledge Hiding

Workplace incivility is defined as “*low-intensity interpersonal mistreatment enacted with ambiguous intent to harm the target*” (Andersson and Pearson, 1999). Employees who experience uncivil behaviors within organizational circuits are likely to develop negative emotional reactions (Weiss and Cropanzano, 1996) leading toward harmful consequences.

The negative consequences of incivility have been documented by previous researchers on employee attitudes and behaviors in the shape of low organizational commitment (Lim and Teo, 2009), decreased job satisfaction (Miner-Rubino and Reed, 2010), uncivil behaviors (Penney and Spector, 2005), higher level of absenteeism (Sliter et al., 2012), and decreased citizenship behavior (Taylor et al., 2012). Broadly knowledge hiding is categorized in three dimensions, Evasive, Playing dumb, and Rationalized knowledge hiding (R-KH; Connelly et al., 2012). Evasive KH is when the knowledge provider tries to misguide the seeker with some erroneous information, while in the case of Playing dumb KH tactics, knowledge provider tends to hide knowledge or information by portraying that he/she does not possess what the knowledge seeker is requesting. R-KH is when the knowledge provider gives justifications to withhold information. However, recently scholars have also proposed other dimensions of knowledge hiding i.e., bullying hiding (Yuan et al., 2020), which denotes a situation where a knowledge provider adopts a harsh and offensive manner to discourage the knowledge seeker from questioning them as a means of protecting their “knowledge power.”

In response to incivility, individuals at the workplace may intentionally withhold knowledge by pretending that they do not have access or awareness of the relevant knowledge/information (Irum et al., 2020). More specifically, when requested for information, individuals may simply choose to respond with false information (Connelly et al., 2012). Past studies indicate that mistreatment at the workplace is associated with knowledge hiding behavior (playing dumb behavior; Zhao et al., 2016). Furthermore, individuals may withdraw from showing helping behaviors to others (citizenship behaviors) as a response to uncivil and hostile treatment (Zellars et al., 2002). Additionally, incivility can trigger negative emotions among victims, which can drive them to take revenge by withholding access to specific information requested by fellow beings by saying that they do not possess or have access to requested knowledge. Simply, playing dumb might be a reasonable choice for victims of incivility under such circumstances. Hence, it is hypothesized that:

*H1*: Workplace incivility triggers employees to hide knowledge.

Rumination is a method of coping with the negative mood that involves self-focused attention (Lyubomirsky and Nolen-Hoeksema, 1993), and it is directly or indirectly related to physiological facts which are used to develop some negative thoughts (Siegle et al., 2003). This lack of concentration usually evolves due to the negative events occurring at workplace in the shape of incivility. Past studies indicate that majority of the workers are not being able to shut the work even after work (Gallie et al., 1998). Thus, employees who are badly affected by rumination will isolate themselves, and in extreme cases, they can withdraw from the helping behaviors, thus reducing the access toward knowledge by portraying that they do not have the required knowledge (Playing dumb KH).

In rumination mostly, it is stated that the ruminator deep down has a strong desire to take revenge because of his clashes (e.g., I want to see him in miserable condition or get hurt or increased in aggression; McCullough et al., 2001). Work-related rumination rate remains high if the environment of job or occupation demanding more emotional and mental power, such as teaching profession (Aronsson et al., 2003; Cropley et al., 2006), and they suffer from job strain due to which they take a long time to unwind the previous work-related issues (Cropley and Purvis, 2003).

Rumination is known as the main cause of depression (Roley et al., 2015), and most researches predict that there is a strong relationship between different types of emotional disorders and rumination (Kong et al., 2015), thus forcefully indulging the individuals to stop sharing knowledge by portraying they do not have required knowledge. Thus, based on the available literature, it can be assumed that:

*H2*: Rumination is positively associated with knowledge hiding in academic staff.

*H3*: Rumination mediates the relationship between workplace incivility and knowledge hiding in academic staff.

### Workplace Incivility, Interpersonal Distrust, and Knowledge Hiding

Distrust has been less studied as compared to trust (Kim et al., 2009; Kramer and Lewicki, 2010). To a great extent, the functions of distrust are largely ignored. The general deficiency of distrust studies might be the aftereffect of the early supposition that distrust was something contrary to trust (i.e., distrust was an absence of trust). Scholars of trust have discovered that distrust and trust are not two closures of a similar continuum; rather, they are different ideas (Lewicki et al., 2006). High distrust is not equivalent to low trust. Therefore, both distrust and trust include assurance and certainty, and distrust speaks to high assurance/trust in negative desires, while trust speaks to high assurance/trust in inspirational desires.

The past studies conclude that distrust usually results in incredulity, disbelieve, or even duplicity\which makes teamwork difficult (Cahill et al., 2003), due to which the effectiveness of the organization is decreased (Levi et al., 2004). Additionally, interpersonal distrust can decrease representatives’ ability to take part in citizenship behaviors or simply helping behaviors. This state of affairs makes the workplace unfriendly and discourages helping behaviors. Interpersonal distrust additionally intercedes the connection between objective incivility and workplace avoidance (Scott et al., 2013) which can trigger employees to deny the knowledge-seeking requests of fellow beings by portraying that they do not have access to specific knowledge.

Interpersonal distrust in the working environment is a desire for hurtful, unfriendly, or other negative results based on experience and is joined by negative feelings and expectations to keep away from those results. There are two potential reasons why workers distrust in specific circumstances. On one side, workers may realize that the organization would not make a proper move to secure its workers when the representative becomes the target of workplace violence (for example, system distrust). On the opposite side, representatives can create learning and feeling toward a subordinate that “He/She is not a responsible individual to have in a working group and he/she generally show incivility to other partners” (e.g., personal distrust).

Interpersonal relationships play an essential role in social exchanges, and past studies indicate that lack of strong personal relationships at the workplace promotes knowledge hiding (Butt and Ahmad, 2020). Thus, the prevalence of interpersonal distrust at the workplace describes a lack of good relationships which harms mutual trust and respect and encouraging the individuals to hide knowledge in organizational circuits (Labafi, 2017). Based on the above arguments, it can be hypothesized that:

*H4*: Workplace incivility is positively associated with Interpersonal distrust in academic staff.

*H5*: Interpersonal distrust is positively associated with knowledge hiding in academic staff.

*H6*: Interpersonal distrust mediates the relationship between workplace incivility and knowledge hiding in academic staff.

## Materials and Methods

### Participants

Participants were recruited through a cross-sectional research design, and data were collected from the academic staff of higher education institutes of public sector in Pakistan. In this regard, those faculty members were approached who were supervising research students of various degree programs at the time of data collection. Higher education institutes are signified for knowledge (Millar et al., 2016); thus, knowledge hiding in higher education institutes can block the flow of knowledge that will eventually influence the individual and organizational performance (Tangaraja et al., 2015).

### Procedure

In a cross-sectional researches issue of a common method, biases can prevail due to self-reported measures which are likely to influence the predictive capability of the findings (Ng and Feldman, 2013); thus to address this issue, we employed various measures. It was ensured to the respondents that collected data will be used only for educational research and the issue of anonymity will be maintained; in addition, we reversed the coding of some items to reduce monotonic responses from the participants (Malhotra et al., 2006). Moreover, data were collected in two waves to avoid common method biases; during the first wave, questions were asked from the respondents regarding their demographic characteristics and exposure to workplace incivility along with rumination. Participants were approached through personal and professional contacts, and to match the responses at time-1 and time-2, the questionnaires were coded. Keeping in view the cross-sectional nature of the study, a sample size of 400 was considered, on the recommendation of Krejcie and Morgan (1970) as 374 sample size is sufficient where the population is unknown. Initially, 400 questionnaires were floated and 355 completed filled responses were received back, while in the second wave, those respondents were approached for data collection who have completely filled questionnaires in the first wave. The time interval between the two waves was 6 weeks. In the second wave, questions related to distrust and knowledge hiding behavior were asked from the respondents. At this stage, 323 questionnaires were received back out of which 285 were completely filled and which were considered for final data analysis. Thus, this time lag double phase data collection helped to reduce potential common method biases (Podsakoff et al., 2003; Chang et al., 2010). According to Podsakoff et al. (2003), time lag during data collection should not be too long nor too short because if the time lag between variables is too short or too long, it can hide relations among study variables artificially (Babalola et al., 2019). Thus, a time lag of 6 weeks offers best choice for time lag data (Walumbwa and Schaubroeck, 2009; Babalola et al., 2019).

### Measures and Demographic Characteristics

Likert scale based on five-point was followed to record the perception of respondents regarding study constructs. The independent variable of this study, that is, workplace incivility, was measured through eight items scale designed by Cortina et al. (2001) on a range of every day (5) to twice in a year (1). Sample items include “Someone at workplace ignored or excluded you from professional circle.” The first mediating variable of this study, that is, rumination, is assessed through five items, although the original scale covers three dimensions of rumination (Affective Rumination, Detachment, and Problem-solving pondering); however, this study anticipated the only one dimension of rumination, that is, affective rumination. To access the perception of the respondent, regarding interpersonal distrust, we use five items of interpersonal distrust developed by Cook and Wall (1980). Sample items include “If I got in difficulties at work, I know my colleagues would not try to help me out” and “I cannot trust the people I work with to lend me a hand if I needed it.” The dependent variable knowledge hiding is comprised of three dimensions, evasive knowledge hiding, rationalized knowledge hiding, and playing dumb (Connelly et al., 2012); however, this study conceptualized one dimension of knowledge hiding, that is, playing dumb through four items adopted by Connelly et al. (2012). Sample questions include “I come to an agreement to help him/her, but I gave him or her the wrong information,” “I suggest him/her some other information as an alternative of what he/she really needs,” and “I pretend that I do not identify the information.” This scale has been used by researchers in the past and most recently utilized by Syed et al. (2021). Demographic characteristics of respondents indicate that most of the respondents were male (58%), while female participants constitute 42%. Similarly, most of the respondents hold Doctoral Degree (71%), while others have 18 years of education (29%). According to the age, brackets the majority of the respondents have an age of more than 30 years (74%), while 26% of respondents have age less than 30 years.

### Statistical Analysis

This study anticipated a structural equation modeling (SEM) approach keeping in view the more complex nature of study constructs (Chin, 1998; Vinzi et al., 2010; Hair et al., 2014). In this regard, Smart PLS provides an alternative approach to CB-SEM. Moreover, theory in the case of knowledge hiding is under development; thus, using PLS-SEM for the explanation of variance was the best available option (Hair et al., 2016). Finally, PLS-SEM deals very well with the non-normal data (Hair et al., 2016).

## Results and Discussion

Structural equation modeling is evaluated based on the measurement and structural models. Firstly, the measurement model was evaluated based on reliability and validity (Hair et al., 2016). All the indicators of reliability were within the acceptable range (see Table 1). Hence, alpha, rho-a, and CR values in the case of workplace incivility were 0.87, 0.903, and 0.904, respectively. Similarly, in the case of mediating and dependent variables, all indicators of reliability were within the acceptable range.

**Table 1:** Reliability and convergent validity of the constructs.

Constructs	Indicator	Indicator reliability	VIF	Alpha	rho-A	Composite reliability	AVE
Workplace incivility	WI2	0.713	3.026	0.87	0.903	0.904	0.613
	WI3	0.729	1.660				
	WI4	0.911	3.962				
	WI6	0.858	3.198				
	WI7	0.692	2.818				
	WI8	0.772	2.300				
Interpersonal distrust	ID2	0.772	1.542	0.823	0.827	0.88	0.654
	ID3	0.788	1.745				
	ID4	0.864	2.169				
	ID5	0.807	1.697				
Rumination	RM1	0.911	3.793	0.918	0.928	0.942	0.902
	RM3	0.830	2.482				
	RM4	0.919	4.228				
	RM5	0.920	4.381				
Knowledge hiding behavior	KH1	0.627	1.230	0.728	0.749	0.831	0.555
	KH2	0.766	1.929				
	KH3	0.717	1.407				
	KH4	0.853	2.171				

While assessing discriminant validity, two mostly used criteria were followed, that is, Fornell and Larcker (1981) criterion (Chin, 2010) and HTMT (Hair et al., 2011). Both the criteria were met as the square root of the AVE of each construct was large enough from correlations among constructs.

While in reflective measurement models, convergent validity is assessed through outer loadings and AVE (Mela and Kopalle, 2002; Hair et al., 2016). Firstly, items with poor loading (<0.40) were located and were dropped from further analysis. From the construct workplace incivility, two items (WI-1 and WI-5) were dropped due to loading below the threshold value (0.708), while ID-1 from interpersonal distrust and R-2 from the construct Rumination were dropped due to poor outer loadings. No item was dropped from the knowledge hiding behavior. However, WI-7 was retained despite low loading because AVE of workplace incivility was greater than 0.50 (Hair et al., 2016). Similarly, KH-1 was also retained in this regard despite the low outer loading (see Table 1).

### Assessment of Structural Model

A bootstrapping procedure based on 5,000 randomly drawn subsamples was followed to evaluate the structural model (Hair et al., 2016). In this regard, firstly, coefficient of determination alternatively known as a measure of predictive accuracy was checked (see Table 2). Here, 65% variation in knowledge hiding was observed due to the combined effect of workplace incivility, interpersonal distrust, and rumination, while workplace incivility indicates 5 and 16% variation in interpersonal distrust and rumination, respectively. Similarly, predictive relevance was assessed based on Q-Square, for which the value of Q-Square must be higher than zero. Thus, predictive relevance was also observed as values of Q-Square were higher than zero for all the endogenous constructs of this study (Hair et al., 2013). For the assessment of the issue of multi-collinearity, VIF values for both, the inner and outer model, were scrutinized (Mela and Kopalle, 2002), and all these values were under the acceptable limit of +5 (Tables 1 and 3).

**Table 2:** Fornell and Larcker (1981); Criteria, HTMT, R-square, and Q-square.

Construct	Interpersonal distrust	Knowledge hiding	Rumination	Workplace incivility	R-square	R-square Adj:	Q-square
Interpersonal distrust	***0.808***	0.704	0.295	0.249	0.050	0.046	0.029
Knowledge hiding	0.525	***0.745***	0.824	0.599	0.654	0.651	0.355
Rumination	0.260	0.703	***0.896***	0.435	0.158	0.155	0.123
Workplace incivility	0.223	0.494	0.397	***0.783***	-	-	-

Bold and Italic values are square root of AVE of respective construct.

**Table 3:** Inner model VIF.

Construct	Interpersonal distrust	Knowledge hiding	Rumination	Workplace incivility
Interpersonal distrust	-	1.093	-	-
Knowledge hiding	-	-	-	-
Rumination	-	1.233	-	-
Workplace incivility	1.000	1.210	1.000	-

Table 4 illustrates the direct, indirect, and total paths. This indicates that path estimates among the study constructs are significant at *p* < 0.05. Path coefficients between workplace incivility and knowledge hiding have been found positive and significant at *p* < 0.05, indicating that workplace incivility triggers individuals to hide knowledge when they experience incivility at the workplace. Similarly, workplace incivility has been found as a predictor of interpersonal distrust at the workplace. Individuals experiencing incivility at the workplace tend to show distrust regarding their fellow beings (co-workers, supervisors, and subordinates). Moreover, it has been found that employees experience a lack of concentration (rumination) when they went through harsh situations at the workplace. State of rumination might be due to the feelings of shame and humiliation resulted due to incivility at the workplace. In the case of distrust and knowledge hiding behavior, it has been proved statistically that employees with the perception of distrust tend to hide knowledge. A similar pattern of results has been observed for the employees who went through rumination. Interesting findings of this study indicate that impact of rumination on knowledge hiding is stronger as compared to other study constructs (coefficient = +0.534), while coefficients for interpersonal distrust and workplace incivility have been found weaker as compared to rumination.

**Table 4:** Direct, indirect, and total paths.

Direct path estimates	Path			
	*Beta*	*SD*	*t*	*p*
Interpersonal distrust ➔ Knowledge hiding	0.339	0.059	5.808	0.00
Rumination ➔ Knowledge hiding	0.534	0.070	7.622	0.00
Workplace incivility ➔ Interpersonal distrust	0.227	0.063	3.526	0.00
Workplace incivility ➔ Knowledge hiding	0.206	0.041	4.991	0.00
Workplace incivility ➔ Rumination	0.402	0.059	6.722	0.00
Indirect path estimates				
Workplace incivility ➔ Interpersonal distrust ➔ Knowledge hiding	0.077	0.027	2.832	0.00
Workplace incivility ➔ Rumination ➔ Knowledge hiding	0.214	0.040	5.263	0.00
Total path estimates				
Interpersonal distrust ➔ Knowledge hiding	0.339	0.059	5.808	0.00
Rumination ➔ Knowledge hiding	0.534	0.070	7.622	0.00
Workplace incivility ➔ Interpersonal distrust	0.227	0.063	3.526	0.00
Workplace incivility ➔ Knowledge hiding	0.498	0.045	11.02	0.00
Workplace incivility ➔ Rumination	0.402	0.059	6.722	0.00

**Figure 1:**
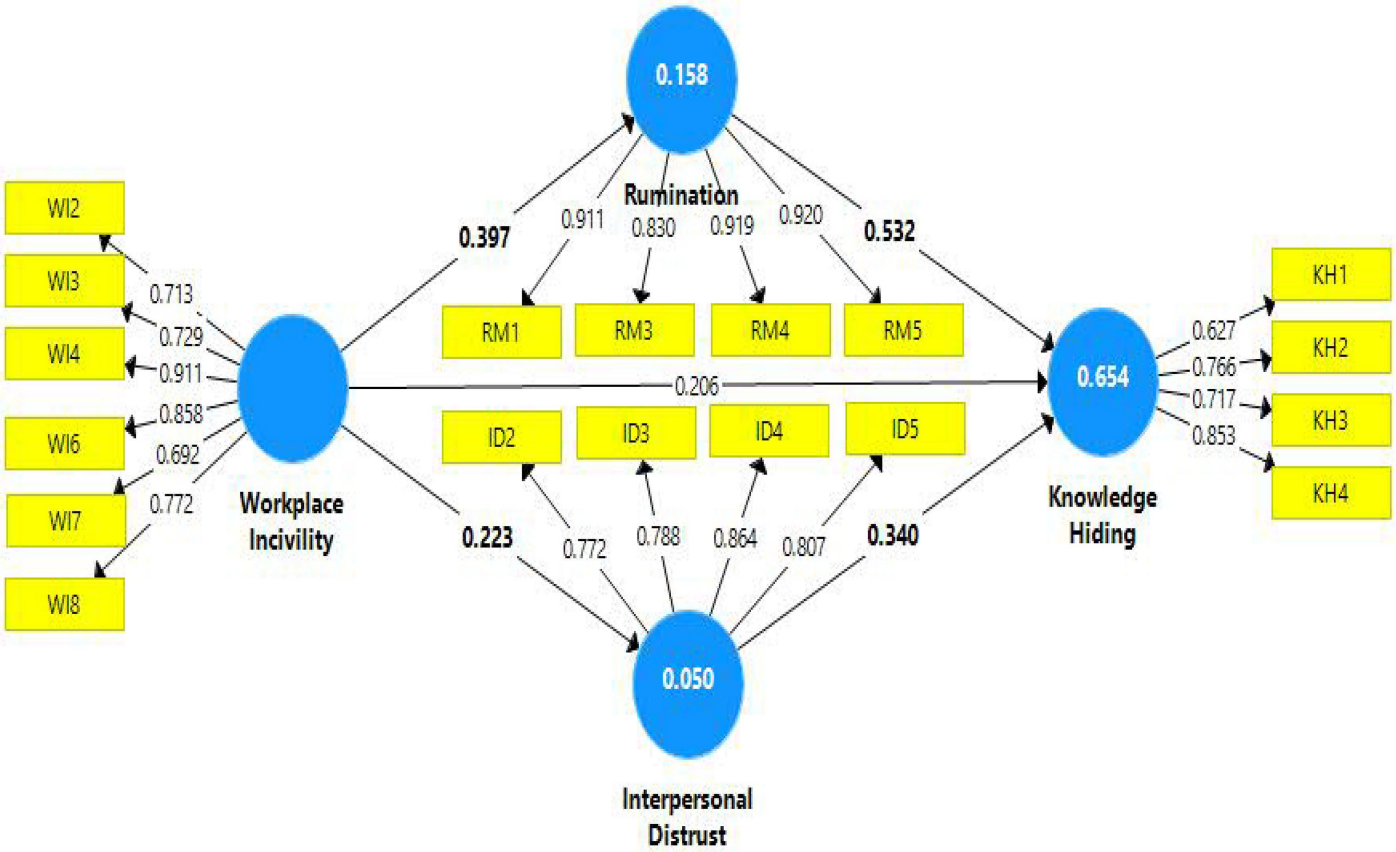
Path diagram.

In the case of mediation analysis, this study anticipated a newly synthesized approach based on variance accounted for (VAF; Hair et al., 2016). In this case, the indirect effect is divided through total effect and the outcome value indicates the nature of mediation, either it is partial, full, or no mediation. In case of path *Workplace Incivility ➔ Rumination➔ Knowledge Hiding*, partial mediation has been observed as the VAF value for this path is 43%. While in the case of *Workplace Incivility ➔ Interpersonal Distrust➔ Knowledge Hiding*, value of VAF has been observed slightly lower than 20% which indicates no mediation. Although the paths between predictor to the mediator and from mediator to outcome variable, in this case, have been found statistically significant, however according to the calculation of VAF, there is no mediation (Figure 1; Table 5).

**Table 5:** Mediation analysis.

Path	Indirect effect	Total effect	VAF
Workplace incivility ➔ Interpersonal distrust➔ Knowledge hiding	0.077	0.498	15%
Workplace incivility ➔ Rumination➔ Knowledge hiding	0.214	0.498	43%

Empirical findings of this study indicate that individuals as a response to workplace incivility fall into rumination. Previous studies indicate that rumination is linked with misleading information. So, developing a rumination in response to incivility further triggers employees to think more negative thoughts rather than remembering positive thoughts (Lyubomirsky et al., 1998). Thus, feelings of humiliation and shame generated as an outcome of incivility trigger employees to think more negatively and persistently about past, present, and future events also. Hence, individuals who ruminate themselves continuously can hide knowledge from fellows (Siegle et al., 2003; Joormann, 2006). Moreover, rumination has been linked to both the victim (Shapiro et al., 2013) and bystanders (Porath et al., 2010) at workplace incivility (Volmer et al., 2012; Demsky et al., 2014; Nicholson and Griffin, 2015); thus, it can hamper the concentration of by-standards too.

Workplace incivility crafts negative feelings/emotions, such as distrust and resentfulness (Lutgen-Sandvik et al., 2007); thus, knowledge hiding phenomena among the academic staff may be impacted by the feelings of distrust and relational connections (Blaug et al., 2007). Interpersonal relationships among the employees may be demolished by mutual distrust (Černe et al., 2014), and broken relations can also trigger knowledge hiding which may also destroy the organizational performance by damaging the mutual understanding among the employees, creation of new thoughts, and implementation of policies (Peng, 2013).

Summing up the findings of this study, it can be concluded that knowledge hiding prevails in academic institutes in response to workplace incivility. Employees tend to hide knowledge when they experience incivility. On the other hand, incivility hampers mutual trust which further triggers employees to hide ideas, information, and knowledge from their fellow workers. In the case of negative emotions, rumination is generated through feelings of humiliation and shame which are generated as an outcome of incivility.

### Study Implications

This study has attempted to add important insights from a theoretical perspective; firstly, this study has made an effort to ascertain the impact of workplace incivility on knowledge hiding in the academic sector (Connelly and Kelloway, 2003; Peng, 2013) by tapping the missing link and investigating how in academic institutes employees tend to cope with workplace incivility (Srivastava et al., 2006). This is the very first study that has linked rumination with knowledge hiding which is a unique contribution of this study. Further, by anticipating the future call of Irum et al. (2020), this study empirically tested the relationship between workplace incivility and knowledge hiding by collecting the sample data from employees of leading public sector organizations in the country.

Moreover, this study explored the role of workplace incivility in relation to knowledge hiding through other behaviors (Irum et al., 2020) by assuming rumination and interpersonal distrust as negative affect in response to workplace incivility. Another contribution of this study is that those faculty members were approached who were supervising research students of various degree programs at the time of data collection. Because higher education institutes are signified for knowledge (Millar et al., 2016), this study indicates that research supervisors can hold knowledge which can hamper the learning process of research students. Thus, knowledge hiding in higher education institutes can block the flow of knowledge that will eventually influence the individual and organizational performance (Tangaraja et al., 2015) and warrants that research students can face problems in learning under hostile and uncivilized academic environments.

Findings of this study supported the premise that employees at the workplace try to cope with negative events (through resource gaining) when the demands at job are high, workplace incivility being a negative phenomenon needs higher resources, while job demands are very high and employees tend to develop negative emotions in the shape of interpersonal distrust by following the exchange mechanism of socio-economic benefits (Blau, 1964). Moreover, when demands at job are high due to workplace incivility, employees may result in higher exhaustion levels and thus falling into rumination (Schaufeli and Bakker, 2004). In this case, this study endorses the premise of job demands-resources (JD-R) model (Bakker and Demerouti, 2007). From a practical point of view, this study posits that workplace incivility triggers knowledge hiding which has the potency to hamper the individual and organizational performance, thus warranting a mechanism to curb workplace incivility through policies and procedures. Previous studies indicate that nonfinancial rewards can reduce knowledge hiding, so providing nonfinancial rewards to the faculty members can provide an edge in academia to promote knowledge sharing (Zhang and Min, 2021).

### Limitations and Future Directions

Frist limitation of this study is associated with its cross-sectional nature which does not permit to induce a cause and effect relationship, so the nature of relationships covered in this study can be generalized but with care. Secondly, the sample size in this study is not large enough, so studies with larger sample sizes can provide deeper insights. Respondents in this study were from public sector universities; in the future, obtaining the response from both public and private sector academic staff surely will provide a more vivid picture because workplace incivility is a more common phenomenon that can be found in all cultures and organizations (Cortina et al., 2001). In this study, incivility has been taken as a one-dimensional concept; thus, adding other attributes of incivility, such as downward, upward, or lateral incivility, can provide deeper insights in the future.

The concept of distrust as negative effect (Lumineau et al., 2015) in team works can make working more difficult due to disbelieving and duplicity (Cahill et al., 2003); thus, investigating distrust along with workplace incivility and knowledge hiding in teams can provide important avenues for future research. Due to distrust, the effectiveness of the organization is decreased (Levi et al., 2004); thus, investigating distrust with creativity and knowledge hiding will also be a good future direction.

Previous literature indicates that knowledge hiding has negative consequences for the project team, and it hampers performance; hence, investigating knowledge hiding phenomena in various teams (e.g., sales team) can be a potential future call (Chatterjee et al., 2021). Moreover, considering the hazardous nature of workplace incivility, future studies can also anticipate moderating phenomena, such as Islamic work ethics to mitigate the negative consequences of workplace incivility (Murtaza et al., 2016). Positive leadership styles can deal with the knowledge hidings behavior very comfortably; thus, considering positive leadership styles, such as ethical leadership style, can also be a good choice in future studies. The way individuals respond toward incivility varies with gender (Cortina and Magley, 2009); thus, gender differences can provide a different approach to how males and females will respond to knowledge requests under an uncivilized environment due to the submissiveness nature (Pearson and Porath, 2005). Thus, considering gender as moderating variable could be a potential future research direction.

Other factors which have the potency to reduce the knowledge hiding behaviors, such as ethical leadership, can be a future avenue for the researchers to investigate knowledge hiding behaviors because ethical leadership can provide a resource gain mechanism, enabling individuals to share knowledge (Anser et al., 2021). Moreover, bridging trust with ethical leadership style can provide important insights for the prediction of knowledge hiding behaviors at the workplace because the element of and integrity and honesty shown by ethical leaders promotes trust culture and thus encouraging the individuals to cater for the knowledge-seeking calls of their co-workers (Anser et al., 2021).

It is further added that incivility at workplace creates the job stress and activates a repetition of thoughts about work which can also affect very badly the quality of someone sleep (Oore et al., 2010; Bayne, 2015; Holm et al., 2015); thus, considering the quality of sleep in future studies is recommended. This study has anticipated only one dimension of rumination (affective rumination); in the future, adding other two dimensions of rumination, that is, problem-solving pondering and detachment, can provide important dimensions. Similarly, this study only considered the one dimension of knowledge hiding, that is, Playing Dumb, so considering other dimensions of knowledge hiding behavior would also be an interesting area of research (Connelly et al., 2012) because literature provides evidence that senior managers tend to hide knowledge from their juniors as they perceive that if they share knowledge with them, chances of their replacement will increase (i.e., rationalized knowledge hiding; Butt, 2021).

## Data Availability Statement

The raw data supporting the conclusions of this article will be made available by the authors, without undue reservation.

## Author Contributions

All authors contributed to data analysis, drafting or revising the article, have agreed on the journal to which the article will be submitted, gave final approval of the version to be published, and agree to be accountable for all aspects of the work.

## Conflict of Interest

JS was employed as Organization Development Manager, by Human Resource Center, Beijing Huaxia Lihong Commodity Inspection Co., Ltd., China.

The remaining authors declare that the research was conducted in the absence of any commercial or financial relationships that could be construed as a potential conflict of interest.

## Publisher’s Note

All claims expressed in this article are solely those of the authors and do not necessarily represent those of their affiliated organizations, or those of the publisher, the editors and the reviewers. Any product that may be evaluated in this article, or claim that may be made by its manufacturer, is not guaranteed or endorsed by the publisher.
